# Photonics based on carbon nanotubes

**DOI:** 10.1186/1556-276X-8-300

**Published:** 2013-06-26

**Authors:** Qingyuan Gu, Maud Gicquel-Guézo, Slimane Loualiche, Julie Le Pouliquen, Thomas Batte, Hervé Folliot, Olivier Dehaese, Frederic Grillot, Yann Battie, Annick Loiseau, Baolai Liang, Diana Huffaker

**Affiliations:** 1 FOTON, UMR CNRS, 6082, INSA, avenue des Buttes de Coësmes CS 14315, 35043 Rennes Cedex, France; 2 Télécom ParisTech, Ecole Nationale Supérieure des Télécommunications, Laboratoire CNRS LTCI, 46 rue Barrault, 75634 Paris Cedex 13, France; 3 LCP-A2MC, Institut de Chimie Physique et Matériaux (ICPM), 1 boulevard Dominique François Arago, 57070 Metz Technopôle, France; 4 LEM, ONERA, BP72, 29 avenue de la Division Leclerc, 92322 Châtillon Cedex, France; 5 Electrical Engineering Department, University of California at Los Angeles, Los Angeles, CA 90095, USA

**Keywords:** Single-walled carbon nanotube (SWCNT), Photonics, Photoluminescence (PL), Saturable absorber (SA), Multiple quantum well (MQW), Laser

## Abstract

Among direct-bandgap semiconducting nanomaterials, single-walled carbon nanotubes (SWCNT) exhibit strong quasi-one-dimensional excitonic optical properties, which confer them a great potential for their integration in future photonics devices as an alternative solution to conventional inorganic semiconductors. In this paper, we will highlight SWCNT optical properties for passive as well as active applications in future optical networking. For passive applications, we directly compare the efficiency and power consumption of saturable absorbers (SAs) based on SWCNT with SA based on conventional multiple quantum wells. For active applications, exceptional photoluminescence properties of SWCNT, such as excellent light-emission stabilities with temperature and excitation power, hold these nanometer-scale materials as prime candidates for future active photonics devices with superior performances.

## Background

Future technologies in photonics emerge ideally from research studies revealing systems with greater performance/cost ratio, as well as more flexible technological orientations with easier manufacturing processes. Single-walled carbon nanotube (SWCNT)-based photonics technology is becoming a reality as commercial photonics solutions include SWCNT-based devices [1]. A large number of studies on SWCNT nonlinear excitonic optical properties for saturable absorption (SA) applications in mode-locking fiber lasers have been reported [2-4]. Nevertheless, the literature on SA applications for SWCNT-based ultrafast optical switching stays poor in number. Conventional SA based on doped multiple quantum wells (MQW) requires expensive growth technologies and complex process of doping control [5]. Recently, one kind of cheaper and easier-to-fabricate SA based on carbon nanotubes demonstrated faster switching time, lower saturation fluence, and higher contrast ratio than MQW-SA [6-11].

Moreover, SWCNT-based technology for active applications in optical networking ever requires research studies, as no SWCNT-based nanolaser has yet been demonstrated. Light emission of SWCNT surrounded by surfactants in liquid media [12] or individual SWCNT suspended on holders [13,14] has been numerously reported. For applications point of view, with durability requirements, solid SWCNT film on substrates is more convenient, but a few photoluminescence studies on efficient light-emitting SWCNT films are reported up to now. Although photoluminescence of a stretch-aligned SWCNT/SDS/gelatin dried film was already reported in 2005 [15], the low concentration of SWCNT hinders practical applications. Photoluminescence of SWCNT layer deposited on quartz and embedded SWCNT in polymer film are demonstrated in [16]. Recently, an important step toward SWCNT-based laser was reported by Gaufres et al. [17], as optical gain in poly(9,9-di-n-octylfluorenyl-2,7-diyl) (PFO)-wrapped semiconducting single-walled nanotube (s-SWNT) was reported. The same research team presented the integration of PFO-wrapped s-SWNT in silicon photonic structures and demonstrated experimentally its light emission in silicon waveguides [18]. Another step has been held by Mueller et al., as they reported electrically driven light emission from aligned SWCNT between two electrodes [19]. In conclusion, the research orientation of SWCNT photoluminescence is gradually advancing from liquid state to solid state, toward light-emitting diodes and laser applications.

Here, we present our work on SWCNT optical properties for passive as well as for active photonics applications in optical networking. We first directly compare SWCNT with MQW absorption nonlinearities, aiming at demonstrating the huge potential of SWCNT-based optical devices for saturable absorption applications as an easier-process and lower-cost efficient solution than conventional semiconductor MQW [10,11]. This work highlights the interest for future photonics to benefit from larger one-dimensional (1D) excitonic nonlinearities in SWCNT than 2D in MQW. Secondly, thanks to SWCNT photoluminescence characterizations, we show a particular behavior of SWCNT film light emission on Si substrate with varying incident powers, as well as over temperature ranging from 77 K to room temperature, as no obvious wavelength shift is observed in both cases. This high stability of SWCNT light-emission energy distinguishes them strongly with any other semiconductor nanomaterials, which are ruled by Varshni's law [20]. This behavior confers a special great interest to SWCNT for new photonics sources with high stability over wide operating temperature range.

## Methods

### Preparation of SWCNT samples

Two types of SWCNT samples were prepared from raw HiPCO SWCNT (purchased from Unidym, Sunnyvale, CA, USA): bundled SWCNT (B-SWCNT) and SWCNT surrounded by micelles (M-SWCNT). For B-SWCNT sample preparation, raw SWCNT powder was initially inserted in N-methyl pyrrolidone solution and sonicated for 1 h; SWCNT suspension was then nitrogen brushed on a heated glass substrate in order to evaporate the solvent. This simple process holds to obtain a dried film of SWCNT in bundles, which has already been structurally analyzed by Raman spectroscopy and scanning tunneling microscopy [11] For M-SWCNT way, 10 mg of pristine SWCNT powder was added to 20 ml of 2%-sodium-cholate water solution, then sonicated for 1 h, and finally centrifuged at 25,000×*g* for 1 h; the upper suspension layer was dropped on a glass substrate, leading to a few microns-thick SWCNT film. We already reported the linear absorption spectra of both samples in [10], which indicate that the SWCNT first excitonic transition energies are suitable for 1,550-nm-window photonics applications.

## Results and discussion

### Comparison of SWCNT and MQW nonlinear optical properties for passive photonics applications: pump-probe experiments

**Figure 1:**
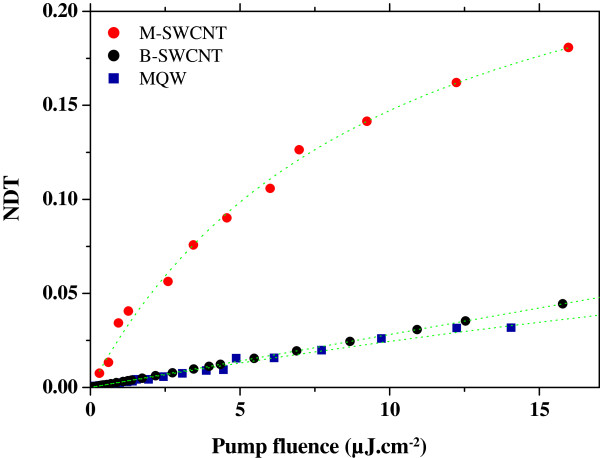
NDT for M-SWCNT, B-SWCNT, and MQW as a function of incident pump fluence at 1550-nm excitation wavelength.

In order to compare SWCNT with MQW optical property performances for saturable absorption and optical switching applications, pump-probe experiments are performed at 1,550 nm with femtosecond optical excitation, and probe pulses originated from an optical parametric oscillator. Details of the experimental setup are provided in [10]. We already demonstrated the ultrafast absorption dynamics of SWCNT in direct comparison with MQW [7] and pointed out the B-SWCNT faster recovery time of absorption dynamics as a great asset of these 1D nanomaterials for ultrafast photonics. Another important key parameter for SA applications is the amplitude of SA nonlinearities, which are characterized by such pump-probe experiments, thanks to the measurement of normalized differential transmission (NDT), defined as NDT = Δ*T*/*T*_0_ = (*T* - *T*_0_)/*T*_0_, where *T*_0_ and *T* are the transmission of the probe at very low and high pump excitation fluences, respectively. NDTs for B-SWCNT, M-SWCNT, and MQW as a function of incident pump fluence at 1550-nm excitation wavelength are demonstrated in Figure 1. Whereas, B-SWCNT and MQW NDTs are closely the same; for a given incident pump fluence, the amplitude of M-SWCNT NDT is clearly greater than B-SWCNT and MQW NDTs (six times greater at 10 μJ cm^-2^, for example). This enhancement of 1D excitonic nonlinearities in M-SWCNT is associated with a reduction of tube-tube interactions, thanks to micelles environment of SWCNT, and contributes to better expected performances of SWCNT-based devices for passive photonics applications. In addition to fast response time and strong nonlinearity as key requirements for nonlinear materials, the power consumption has to be as low as possible, for general energy consumption control in future photonics [3]. The power consumption is related to the input fluence required for inducing a switching phenomenon of nonlinear materials, called saturation fluence *F*_S_. Using the semiempirical absorption saturation relation [21] of a two-energy level system, NDT can be expressed as NDT = Δ*T*/*T*_0_ = exp[*A*/(1 + *F*_S_/*F*)] - 1, where *A*, *F*, and *F*_S_ are the absorbance, the excitation fluence, and the saturation fluence, respectively. Generally, NDT reflects the quality of regenerative signal: higher NDT, higher quality. Regardless of the absorption *A*, higher NDT demands lower saturation fluence *F*_*S*_. From the adjustments of this NDT analytic expression represented in dotted lines in Figure 1 with experimental curves, we extract *F*_*S*_ values of 9, 70, and 726 μJ cm^-2^ for M-SWCNT, MQW, and B-SWCNT, respectively. These results indicate that M-SWCNT-based photonics devices are expected to consume eight times less than MQW-based and 80 times less than B-SWCNT-based devices. The greater B-SWCNT *F*_S_ value, in comparison with M-SWCNT, is associated with the higher number of nonradiative excitonic relaxation pathways in B-SWCNTs, especially due to charge tunnel transfer from semiconducting to metallic tubes within a bundle [6]. Hence, shorter exciton lifetime in B-SWCNT than in M-SWCNT leads to greater incident energy to saturate B-SWCNT absorption than M-SWCNT absorption.

Finally, M-SWCNT are promising nonlinear materials for efficient, ultrafast, low-cost future passive photonics devices in optical networking with lower power consumption than conventional MQW semiconductors. A further progress to lower power consumption again should be loaded by the alignment of SWCNT in order to favor light-matter interactions. This technological step is in progress.

### Toward active photonics devices: SWCNT photoluminescence experiments

Among the key requirements for light sources in optical networking, emission stabilities with temperature and incident power are of great importance. Also, light emission from SWCNT requires debundling of SWCNT [12], as huge numbers of excitonic nonradiative recombination pathways are available within bundles, thanks to tube-tube contacts, leading to photoluminescence (PL) quenching. Therefore, only M-SWCNT sample studies are suitable for active photonics applications. The preparation of M-SWCNT samples is mentioned above.

Light emission of M-SWCNT is characterized by PL spectroscopy experiments, using continuous-wave excitation laser and InGaAs detector, covering 800- to 1,700-nm wavelength window.

**Figure 2:**
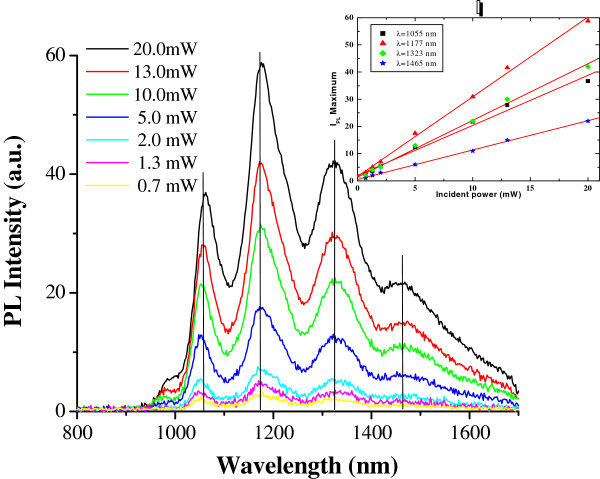
**M-SWCNT photoluminescence (PL) spectra, at 10-mW excitation power and 659-nm excitation wavelength laser. **With temperature ranging from 77 to 300 K. Vertical lines are guides for the eyes.

Figure 2 shows M-SWCNT photoluminescence spectra at room temperature and 659-nm excitation wavelength, under different incident power levels (from 0.7 to 20.0 mW). We observe different light-emission peaks, which are attributed to different SWCNT chiralities. The particular behavior of light-emission M-SWCNT highlighted by these PL spectra is that no obvious emission wavelength shift is observed, whereas incident excitation power changes. Furthermore, PL intensities exhibit a linear dependence (see the inset of Figure 2) on incident power, over the excitation range examined.

**Figure 3:**
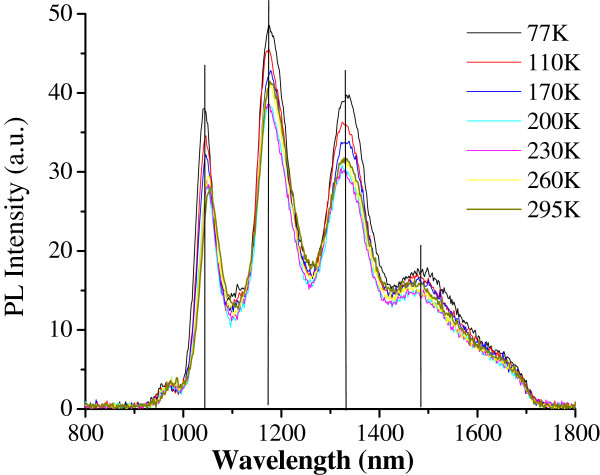
M-SWCNT PL spectra at room temperature and 659-nm excitation wavelength laser under various incident power levels.

Figure 3 reports the evolution of M-SWCNT PL spectra with temperature ranging from 77 to 300 K, at 10-mW excitation power and 659-nm excitation wavelength laser. These spectra are particularly stable with temperature, without any obvious emission wavelength shift and only 20% of PL intensity loss over the whole examined temperature range. This high stability of light-emission wavelength with temperature is in contradiction with the well-known Varshni's law for semiconductor materials [20], which is expressed as *E*_g_ = *E*_0_ - *αT*^2^/(*T* + *β*), where *E*_0_ is the bandgap energy at absolute 0 K and *α* and *β* are material parameter-specific constants.

Although further studies are necessary in order to fully understand the origin of SWCNT light-emission wavelength stabilities with incident power, as well as with temperature, we are firmly convinced that these remarkable light-emission stabilities represent an extraordinary opportunity for SWCNT being a candidate as active materials for future lasers. For practical use, photonics applications require electrically driven active sources; therefore, we aim at combining electrically pumped conventional inorganic semiconductors [22] with SWCNT as light emitters within a same laser cavity, leading to a hybrid laser cavity.

## Conclusions

In summary, we highlight optical properties of SWCNT for future passive as well as active photonics devices. Thanks to a direct comparison with conventional MQW, we show greater nonlinearities and lower required energy for inducing switching phenomenon in M-SWCNT-based saturable absorbers. These performances confer to M-SWCNT's great potential for passive applications for optical switching in optical networking. Further progress should be provided by the alignment of SWCNT, which technological step is in progress.

The results of PL experiments on M-SWCNT indicate exceptional stabilities of light-emission wavelengths with incident excitation power, as well as with temperature. The realization of an electrically pumped hybrid laser, based on SWCNT and conventional inorganic semiconductors of ultrahigh stability, is in progress. In brief, SWCNT demonstrates unique photonics properties for being a promising candidate material of future photonics applications.

## Competing interests

The authors declare that they have no competing interests.

## Authors' contributions

QG participated in the samples preparation and drafted the manuscript. MGG performed the pump-probe measurements and coordinated the manuscript writing. JLP, TB, and YB developed samples preparation methods. HF and FG participated in PL characterizations coordination. BL investigated PL characterizations. OD was in charge of the growth of MQW by molecular beam. SL, DH, and AL contributed to the coordination of all studies. All authors read and approved the final manuscript.
